# Analysis of Antibiotic Exposure and Early-Onset Neonatal Sepsis in Europe, North America, and Australia

**DOI:** 10.1001/jamanetworkopen.2022.43691

**Published:** 2022-11-23

**Authors:** Eric Giannoni, Varvara Dimopoulou, Claus Klingenberg, Lars Navér, Viveka Nordberg, Alberto Berardi, Salhab el Helou, Gerhard Fusch, Joseph M. Bliss, Dirk Lehnick, Nicholas Guerina, Joanna Seliga-Siwecka, Pierre Maton, Donatienne Lagae, Judit Mari, Jan Janota, Philipp K. A. Agyeman, Riccardo Pfister, Giuseppe Latorre, Gianfranco Maffei, Nicola Laforgia, Enikő Mózes, Ketil Størdal, Tobias Strunk, Martin Stocker

**Affiliations:** 1 Clinic of Neonatology, Department Mother-Woman-Child, Lausanne University Hospital and University of Lausanne, Lausanne, Switzerland; 2 Paediatric Research Group, Faculty of Health Sciences, UiT-The Arctic University of Norway, Tromsø, Norway; 3 Department of Pediatrics and Adolescence Medicine, University Hospital of North Norway, Tromsø, Norway; 4 Department of Neonatology, Karolinska University Hospital and Department of Clinical Science, Intervention and Technology, Karolinska Institutet, Stockholm, Sweden; 5 Neonatal Intensive Care Unit, Mother and Child Department, Policlinico University Hospital, Modena, Italy; 6 Division of Neonatology, Department of Pediatrics, McMaster Children’s Hospital, McMaster University, Hamilton Health Sciences, Hamilton, Ontario, Canada; 7 Department of Pediatrics, Women & Infants Hospital of Rhode Island, Warren Alpert Medical School of Brown University, Providence; 8 Biostatistics and Methodology, CTU-CS, Department of Health Sciences and Medicine, University of Lucerne, Lucerne, Switzerland; 9 Department of Neonatology and Neonatal Intensive Care, Medical University of Warsaw, Warsaw, Poland; 10 Service Néonatal, Clinique CHC-Montlegia, Groupe Santé CHC, Liège, Belgium; 11 Neonatology and Neonatal Intensive Care Unit, CHIREC-Delta Hospital, Brussels, Belgium; 12 Department of Paediatrics, University of Szeged, Szeged, Hungary; 13 Neonatal Unit, Department of Obstetrics and Gynecology, Motol University Hospital Prague, Prague, Czech Republic; 14 Department of Pathological Physiology, 1st Medical School, Charles University Prague, Prague, Czech Republic; 15 Department of Neonatology, Thomayer University Hospital Prague, Prague, Czech Republic; 16 Department of Pediatrics, Inselspital, Bern University Hospital, University of Bern, Bern, Switzerland; 17 Neonatology and Paediatric Intensive Care Unit, Geneva University Hospitals and Geneva University, Geneva, Switzerland; 18 Neonatology and Neonatal Intensive Care Unit, Ecclesiastical General Hospital F. Miulli, Acquaviva delle Fonti, Italy; 19 Neonatology and Neonatal Intensive Care Unit, Policlinico Riuniti Foggia, Foggia, Italy; 20 Neonatologia e Terapia Intensiva Neonatale, University of Bari, Bari, Italy; 21 Perinatal Intensive Care Unit, Department of Obstetrics and Gynaecology, Semmelweis University, Budapest, Hungary; 22 Institute of Clinical Medicine, University of Oslo and Oslo University Hospital, Oslo, Norway; 23 Neonatal Directorate, Child and Adolescent Health Service, King Edward Memorial Hospital, Perth, Western Australia, Australia; 24 Department of Pediatrics, Children’s Hospital Lucerne, Lucerne, Switzerland

## Abstract

**Importance:**

Appropriate use of antibiotics is life-saving in neonatal early-onset sepsis (EOS), but overuse of antibiotics is associated with antimicrobial resistance and long-term adverse outcomes. Large international studies quantifying early-life antibiotic exposure along with EOS incidence are needed to provide a basis for future interventions aimed at safely reducing neonatal antibiotic exposure.

**Objective:**

To compare early postnatal exposure to antibiotics, incidence of EOS, and mortality among different networks in high-income countries.

**Design, Setting, and Participants:**

This is a retrospective, cross-sectional study of late-preterm and full-term neonates born between January 1, 2014, and December 31, 2018, in 13 hospital-based or population-based networks from 11 countries in Europe and North America and Australia. The study included all infants born alive at a gestational age greater than or equal to 34 weeks in the participating networks. Data were analyzed from October 2021 to March 2022.

**Exposures:**

Exposure to antibiotics started in the first postnatal week.

**Main Outcomes and Measures:**

The main outcomes were the proportion of late-preterm and full-term neonates receiving intravenous antibiotics, the duration of antibiotic treatment, the incidence of culture-proven EOS, and all-cause and EOS-associated mortality.

**Results:**

A total of 757 979 late-preterm and full-term neonates were born in the participating networks during the study period; 21 703 neonates (2.86%; 95% CI, 2.83%-2.90%), including 12 886 boys (59.4%) with a median (IQR) gestational age of 39 (36-40) weeks and median (IQR) birth weight of 3250 (2750-3750) g, received intravenous antibiotics during the first postnatal week. The proportion of neonates started on antibiotics ranged from 1.18% to 12.45% among networks. The median (IQR) duration of treatment was 9 (7-14) days for neonates with EOS and 4 (3-6) days for those without EOS. This led to an antibiotic exposure of 135 days per 1000 live births (range across networks, 54-491 days per 1000 live births). The incidence of EOS was 0.49 cases per 1000 live births (range, 0.18-1.45 cases per 1000 live births). EOS-associated mortality was 3.20% (12 of 375 neonates; range, 0.00%-12.00%). For each case of EOS, 58 neonates were started on antibiotics and 273 antibiotic days were administered.

**Conclusions and Relevance:**

The findings of this study suggest that antibiotic exposure during the first postnatal week is disproportionate compared with the burden of EOS and that there are wide (up to 9-fold) variations internationally. This study defined a set of indicators reporting on both dimensions to facilitate benchmarking and future interventions aimed at safely reducing antibiotic exposure in early life.

## Key Points

**Question:**

What is the rate of exposure to antibiotics and the incidence of early-onset sepsis (EOS) and sepsis-associated mortality among late-preterm and full-term newborns?

**Findings:**

In this cross-sectional study of 757 979 neonates born in 13 networks from 11 countries, 2.86% received antibiotics during the first postnatal week (range across networks, 1.18%-12.45%). The incidence of EOS was 0.49 cases per 1000 live births (range, 0.18-1.45 cases per 1000 live births), and the EOS-associated mortality rate was 3.20%.

**Meaning:**

Early postnatal antibiotic use was high compared with the rate of sepsis and varied across networks, suggesting it could be lowered safely.

## Teaser

This cross-sectional study compares early postnatal exposure to antibiotics, incidence of early-onset sepsis, and mortality among different networks in high-income countries.

## Introduction

Worldwide, approximately one-quarter to one-third of all hospital-admitted patients are treated with antibiotics.^1,2,3^ The World Health Organization has called for urgent action to avoid an antimicrobial resistance crisis, placing antimicrobial stewardship (AMS) programs at the center of attention to help physicians optimize antibiotic prescription and improve patients’ outcomes.^4^ In addition to increasing rates of antimicrobial resistance,^5,6^ early-life antibiotic exposure disrupts the developing microbiome, which may contribute to numerous diseases later in life, including diabetes, obesity, inflammatory bowel disease, asthma, and allergy.^7^ Furthermore, neonatal antibiotic treatments are associated with mother-newborn separation, longer duration of hospital stay, reduced breastfeeding rates, and increased health care costs.^8^ AMS is, thus, of crucial importance at the start of life.

Antibiotics are the most commonly prescribed medication in neonatal units, and their prompt initiation can be life-saving in neonatal early-onset sepsis (EOS).^9^ However, for late-preterm and full-term neonates, the incidence of EOS has decreased over the last decades.^10^ Limited precision of current diagnostic tools and resulting concern of missing sepsis in conjunction with unchanged management strategies for suspected EOS are the main factors associated with antibiotic overuse in early life.^11,12,13^

Antibiotic exposure within the first postnatal week in late-preterm and full-term neonates along with incidence of EOS have not been quantified at scale internationally. Large data sets are required to compare antibiotic use and monitor time trends in networks and countries using different strategies to prevent and treat EOS, to provide a basis for interventions aiming at safely reducing neonatal antibiotic exposure.^14^ Moreover, standards regarding key indicators are missing for this patient population.^15^ We conducted a large international study to quantify neonatal antibiotic exposure, incidence of EOS, and all-cause and EOS-associated mortality in high-income countries.

## Methods

### Study Design

The Antibiotic Exposure for Suspected Neonatal Early-Onset Sepsis (AENEAS) study is an international, cross-sectional, retrospective study investigating exposure to intravenous antibiotics in the first postnatal week in late-preterm and full-term neonates. Thirteen networks from 11 high-income countries contributed to the study (eTable 1 in Supplement 1).

A network was defined by use of a common strategy for preventing and managing suspected EOS and the ability to provide data on at least 25 000 live births within the 5-year study period. Networks from Europe, North America, and Australia were eligible if they met the expected sample size (eAppendix in Supplement 1) and had the capacity to provide high-quality data on the main study outcomes. Candidate networks were approached on the basis of their record in the field, through the Neonatal Sepsis Trial Network and the European Society for Pediatric Research. The study was approved by the Swiss national ethics committee on human research and by the ethics committees of all participating networks. The need for obtaining written informed consent was waived because the potential difficulties in obtaining consent were considered disproportionate to the low risk and observational nature of the study. This study followed the Strengthening the Reporting of Observational Studies in Epidemiology (STROBE) for Newborn Infection (STROBE-NI) guideline.^16^

### Procedures

We included all neonates born alive at a gestational age of 34 weeks or later between January 1, 2014, and December 31, 2018, and treated for any reason with intravenous antibiotics started within the first postnatal week. There were no exclusion criteria. We recorded the total number of births and neonatal deaths and collected data on network characteristics and guidelines to prevent EOS and manage newborns at risk of EOS (eTable 2 in Supplement 1). Data were extracted from electronic health records, clinical information systems, patient records, databases of microbiology laboratories, and regional and national databases by investigators at each site and transferred into a REDCap database. The principal investigators and statistical team controlled data quality and provided feedback to investigators at each site to optimize data completeness and quality.

### Outcome Measures and Definitions

The primary objective was to quantify antibiotic exposure, incidence of EOS, and mortality in different networks. We classified networks capturing all births within a geographical area as population-based and hospitals or group of hospitals from a single country using the same strategies for prevention and management of newborns at risk of EOS as hospital-based networks.

EOS was defined as culture-proven EOS, by positive blood and/or cerebrospinal fluid (CSF) culture, within the first postnatal week.^17^ Contaminated blood or CSF cultures were defined by growth of bacteria usually considered as contaminants (eg, diphtheroids or *Micrococcus* species) or cultures considered as a contamination by clinicians with a decision to treat with antibiotics for less than 5 days. Cultures with growth of coagulase-negative staphylococci (CoNS) and antibiotic therapy for more than 5 days were considered proven infection. Neonates without proven EOS (no EOS) were those who received antibiotics but did not have a diagnosis of EOS. Indication for antibiotic treatment was obtained from electronic medical records and patients’ records. Neonatal death was defined as death before discharge or death before 28 days for patients who were hospitalized for more than 28 days. EOS-associated mortality was defined as death within 28 days after a positive blood or CSF culture. The cause of death was reported as free text and was then classified into categories (respiratory and/or cardiocirculatory failure, asphyxia, malformation, or other) by the principal investigators.

### Statistical Analysis

The proportion of newborns treated with antibiotics was calculated by dividing the number of newborns receiving at least 1 dose of intravenous antibiotics within the first postnatal week by the number of live births. Duration of antibiotic therapy was defined as calendar days with at least 1 dose of antibiotics. Antibiotic exposure was calculated as the sum of antibiotic days of each treated newborn divided by the number of live births and was reported as the number of antibiotic days per 1000 live births. Incidence of EOS was defined as the rate in all live-born neonates. Incidence was reported for all EOS episodes and for episodes excluding CoNS. Mortality was defined as all events of death for all live-born neonates independent of antibiotic therapy and EOS (overall mortality), and in subgroups (EOS and no EOS). We analyzed the whole cohort and each network separately. We conducted subgroup analyses of data from Stockholm County, Sweden, a large population-based network with the lowest exposure to antibiotics, stratifying data by level of care at place of birth.^18^

We presented descriptive statistics as median (IQR) for continuous variables and as frequencies (percentages) with 95% CIs for categorical variables. We calculated odds ratios (ORs) with 95% CIs using the Mantel-Haenszel method for stratification, as measures of association where binary outcomes were compared between groups. For the comparison of quantitative outcomes between groups, we calculated median differences with 95% CIs. If appropriate, these effect measures were calculated in a stratified manner, using network as strata. Correlations between quantitative metrics were assessed using Spearman correlation coefficient. We performed analyses using R version 4.0.2 (R Project for Statistical Computing). Data were analyzed from October 2021 to March 2022.

## Results

A total of 757 979 late-preterm and full-term neonates were born between January 1, 2014, and December 31, 2018, in participating networks; 21 703 neonates (2.86%; 95% CI, 2.83%-2.90%), including 12 886 boys (59.4%) with a median (IQR) gestational age of 39 (36-40) weeks and median (IQR) birth weight of 3250 (2750-3750) g, were started on intravenous antibiotics within the first postnatal week (Table 1 and Table 2). The median (IQR) duration of antibiotic therapy was 4 (3-6) days for all newborns treated with antibiotics, 9 (7-14) days for those with EOS, and 4 (3-6) days for those without proven EOS (Table 2). The median (IQR) antibiotic exposure was 135 (134-136) antibiotic days per 1000 live births. EOS was diagnosed in 375 neonates, leading to an incidence of 0.49 cases per 1000 live births (95% CI, 0.45-0.55 cases per 1000 live births). Overall, for each case of EOS, 58 neonates were treated with antibiotics, and 273 antibiotic days were administered. More than 95% of antibiotic courses initiated within the first postnatal week were started within the first 3 days. The median (IQR) postnatal age at antibiotic start was 1 (0-2) day in EOS, and 0 (0-1) day in cases without proven EOS (eFigure 1 in Supplement 1). The predominant pathogens identified in blood and CSF cultures were group B streptococci (126 of 375 cultures [34%]), CoNS (65 of 375 cultures [17%]), and *Escherichia coli* (62 of 375 cultures [17%]) (eTable 3 in Supplement 1).

**Table 1.** Main Outcomes in Each Network^a^

Country, region	Type of network	Births, No.	Treated neonates, No. (%)^b^	Duration of antibiotic treatment, median (IQR), d				Antibiotic days, No.^d^	EOS cases, No. (‰)		All-cause deaths, No. (‰)				
				All treated neonates^b^	EOS cases	EOS cases without CoNS^c^	Cases without proven EOS		All EOS cases	EOS cases without CoNS^c^	All neonates	All treated neonates^b^	EOS cases	EOS cases without CoNS^c^	Cases without proven EOS
All networks	NA	757 979	21 703 (2.86)	4 (3-6)	9 (7-14)	10 (7-14)	4 (3-6)	102 486 (135)	375 (0.49)	310 (0.41)	623 (0.82)	239 (1.10)	12 (3.20)	11 (3.50)	227 (1.10)
Population-based networks	NA	408 248	8413 (2.06)	4 (3-6)	8 (6-11)	8 (6-11)	4 (3-6)	37 861 (92)	193 (0.47)	163 (0.40)	212 (0.52)	121 (1.40)	5 (2.60)	4 (2.50)	116 (1.40)
Hospital-based networks	NA	349 731	13 290 (3.80)	4 (3-6)	11 (7-15)	11 (8-15)	4 (3-6)	64 625 (185)	182 (0.52)	147 (0.42)	411 (1.18)	118 (0.89)	7 (3.80)	7 (4.80)	111 (0.80)
Norway^e^	Population-based data	228 199	5767 (2.52)	4 (3-6)	8 (6-11)	8 (6-11)	4 (3-6)	26 273 (115)	143 (0.63)	117 (0.51)	149 (0.65)	82 (1.40)	3 (2.10)	2 (1.70)	79 (1.40)
Sweden, Stockholm County	Population-based data	144 417	1701 (1.18)	4 (3-6)	9 (8-11)	9 (8-11)	4 (3-5)	7792 (54)	42 (0.29)	41 (0.21)	48 (0.33)	32 (1.90)	2 (4.80)	2 (4.90)	30 (1.80)
Central Switzerland	Population-based data	35 632	945 (2.65)	4 (2-5)	10 (9-14)	14 (10-15)	4 (2-5)	3796 (107)	8 (0.22)	5 (0.14)	15 (0.42)	7 (0.90)	0	0	7 (0.70)
Italy, Emilia Romagna	4 Level ≥III hospitals	55 345	931 (1.68)	5 (3-7)	8 (7-14)	9 (8-14)	4 (3-6)	5118 (92)	25 (0.45)	22 (0.40)	22 (0.40)	18 (1.90)	3 (12.00)	3 (13.60)	15 (1.70)
Western Switzerland	3 Level ≥III, 1 level II hospitals	46 169	1303 (2.82)	3 (3-6)	10 (8-14)	11 (8-15)	3 (3-6)	5835 (126)	17 (0.37)	14 (0.30)	52 (1.13)	15 (1.20)	1 (5.90)	1 (7.10)	14 (1.10)
Canada, Hamilton	1 Level ≥III, 2 level II hospitals	44 502	2123 (4.77)	4 (4-7)^f^	12 (9-15)	14 (11-16)	4 (3-7)^f^	10 256 (230)^f^	24 (0.54)	19 (0.43)	92 (2.07)	17 (0.80)	1 (4.20)	1 (5.30)	16 (0.80)
United States, Rhode Island	1 Level ≥III hospital	39 819	963 (2.42)	3 (3-3)	12 (11-16)	12 (11-16)	3 (3-3)	3486 (88)	7 (0.18)	7 (0.18)	17 (0.43)	4 (0.40)	0	0	4 (0.40)
Hungary	2 Level ≥III hospitals	29 648	1285 (4.33)	3 (3-5)	8 (6-11)	11 (8-16)	3 (3-5)	5322 (180)	17 (0.34)	9 (0.30)	33 (1.11)	11 (0.90)	0	0	11 (0.90)
Italy, Apulia	3 Level ≥III hospitals	29 599	1320 (4.46)	7 (6-10)^f^	11 (8-15)	12 (10-16)	7 (6-10)	11 467 (387)^f^	43 (1.45)	35 (1.18)	16 (0.54)	4 (0.30)	0	0	4 (0.30)
Belgium, Wallonia	2 Level ≥III hospitals	28 402	716 (2.52)	4 (4-5)	11 (11-15)	11 (11-15)	4 (4-5)	3397 (120)	11 (0.39)	11 (0.39)	47 (1.65)	6 (0.80)	0	0	6 (0.90)
Czech Republic, Prague	1 Level ≥III, 1 level II hospital	26 985	510 (1.89)	5 (3-6)	7 (7-14)	7 (7-14)	5 (3-6)	2387 (88)	9 (0.33)	7 (0.26)	14 (0.52)	2 (0.40)	1 (11.10)	1 (14.30)	1 (0.20)
Australia, Perth	1 Level ≥III hospital	26 087	3249 (12.45)	3 (3-4)	9 (7-15)	9 (7-15)	3 (3-4)	12 811 (491)	19 (0.73)	16 (0.61)	61 (2.34)	20 (0.60)	0	0	20 (0.60)
Poland, Warsaw	2 Level ≥III hospitals	23 175	890 (3.84)	4 (3-6)	12 (8-11)	15 (8-15)	4 (3-6)	4546 (196)	10 (0.43)	7 (0.30)	57 (2.46)	21 (2.40)	1 (10.00)	1 (14.30)	20 (2.30)

Abbreviations: CoNS, coagulase-negative staphylococci; EOS, early-onset sepsis; NA, not applicable.

^a^ Categorical variables are presented as frequencies (percentages), and continuous variables are presented as median (IQR). Column percentages are presented; percentages are based on available data for each variable.

^b^ Treated neonates refers to neonates treated with intravenous antibiotics during the first postnatal week.

^c^ Refers to number of EOS cases without inclusion of CoNS cases.

^d^ The number in parentheses shows number of antibiotic days per 1000 live births.

^e^ For Norway, data were available from 2015 to 2018.

^f^ Data were missing for 1 patient.

**Table 2.** Demographic and Clinical Characteristics of Infants Treated With Intravenous Antibiotics During the First Postnatal Week^a^

Clinical characteristics	Neonates, No. (%)			OR or median difference (95% CI)
	All (n = 21 703)	Proven EOS (n = 375)	No proven EOS (n = 21 328)	
Sex				
Female^b^	8812 (40.6)	161 (42.9)	8651 (40.6)	1.10 (0.90 to 1.35)^c^
Male	12 886 (59.4)	214 (57.1)	12 672 (59.4)	1 [Reference]
Gestational age, median (IQR), wk	39 (36 to 40)	39 (37 to 40)	39 (36 to 40)	0 (0 to 1)^d^
Birth weight, median (IQR), g	3250 (2750 to 3750)	3250 (2780 to 3785)	3250 (2745 to 3750)	110 (−15 to 230)^d^
Indication of antibiotics^e^				
Suspected infection	14 139 (88.9)	216 (97.3)	13 923 (88.8)	NA
Prophylaxis for urinary tract malformation	185 (1.2)	0	185 (1.2)	
Perioperative prophylaxis	296 (1.9)	0	296 (1.9)	
Other prophylaxis	1289 (8.1)	6 (2.7)	1283 (8.2)	
Postnatal age at antibiotics start, median (IQR), d	0 (0 to 1)	1 (0 to 2)	0 (0 to 1)	0 (0 to 1)^d^
Duration of treatment, median (IQR), d^f^	4 (3 to 6)	9 (7 to 14)	4 (3 to 6)	5 (4 to 6)^d^
Clinical signs^g^				
Present	7951 (75.3)	170 (89.9)	7781 (75)	3.73 (2.24 to 6.33)^c^
Unknown	239 (2.3)	1 (0.5)	238 (2.3)	0.39 (0.05 to 2.96)^c^
Absent	2374 (22.5)	18 (9.5)	2356 (22.7)	1 [Reference]
Deaths	239 (1.1)	12 (3.2)	227 (1.1)	2.81 (1.55 to 5.09)^c^^,^^h^
Postnatal age at death, median (IQR), d^e^	4 (1 to 9)	4 (2 to 10)	4 (1 to 9)	0 (−2 to 2)^d^
Cause of death^e^				
Respiratory and/or cardio-circulatory failure	27 (17.2)	7 (77.8)	20 (14)	NA
Asphyxia	43 (27.4)	0	43 (29.1)	
Malformation	81 (51.6)	2 (22.2)	79 (53.4)	
Other	6 (3.8)	0	6 (4.1)	
Death in relation to sepsis^e^				
Directly related	6 (3.8)	6 (66.7)	0	NA
Indirectly related	5 (3.2)	3 (33.3)	2 (1.4)	
Unrelated	146 (93.0)	0	146 (98.6)	

Abbreviations: EOS, early-onset sepsis; NA, not applicable; OR, odds ratio.

^a^ Categorical variables are presented as frequencies (percentages), and continuous variables are presented as median (IQR). Column percentages are presented; percentages are based on available data for each variable.

^b^ Data are missing for 5 patients without proven EOS. OR is calculated using male neonates as reference.

^c^ Data are OR with 95% CI, stratified across networks.

^d^ Data are median difference with 95% CI, stratified across networks.

^e^ Data on 15 936 of 21 703 neonates (73.4%) treated with antibiotics are presented, including 222 of 375 neonates (59.2%) with proven EOS, and 15 687 of 21 328 neonates (73.6%) without proven EOS, because data were not available for Norway.

^f^ Data are missing for 2 patients without proven infection.

^g^ Data on 10 564 of 21 703 neonates (48.7%) treated with antibiotics are presented, including 189 of 375 neonates (50.4%) with proven EOS, and 10 375 of 21 328 neonates (48.6%) without proven EOS, because data were not available for Norway, Perth, and Hamilton.

^h^ Data are OR (95% CI) for proven EOS (vs no proven EOS), stratified across networks.

The number of deaths for all live births was 622, leading to an all-cause mortality rate of 0.82 per 1000 live births (95% CI, 0.76-0.89 per 1000 live births). The all-cause mortality rate was 1.10% (239 of 21 703 neonates; 95% CI, 0.96%-1.24%) in all neonates treated with antibiotics, 3.20% (12 of 375 neonates; 95% CI, 1.70%-5.50%) in those with EOS, and 1.06% (227 of 21 328 neonates; 95% CI, 0.93%-1.21%) in those without proven EOS. Among EOS cases, mortality was 7.7% (6 of 78 neonates; 95% CI, 2.9%-16.0%) in late-preterm and 2.0% in full-term newborns (6 of 297 neonates; 95% CI, 0.7%-4.3%). Culture-proven EOS accounted for 1.9% of all-cause mortality. Altogether, 1 neonate died from EOS per 63 165 births (12 of 757 979 births; 0.016‰; 95% CI, 0.008‰-0.028‰).

**Figure 1.**
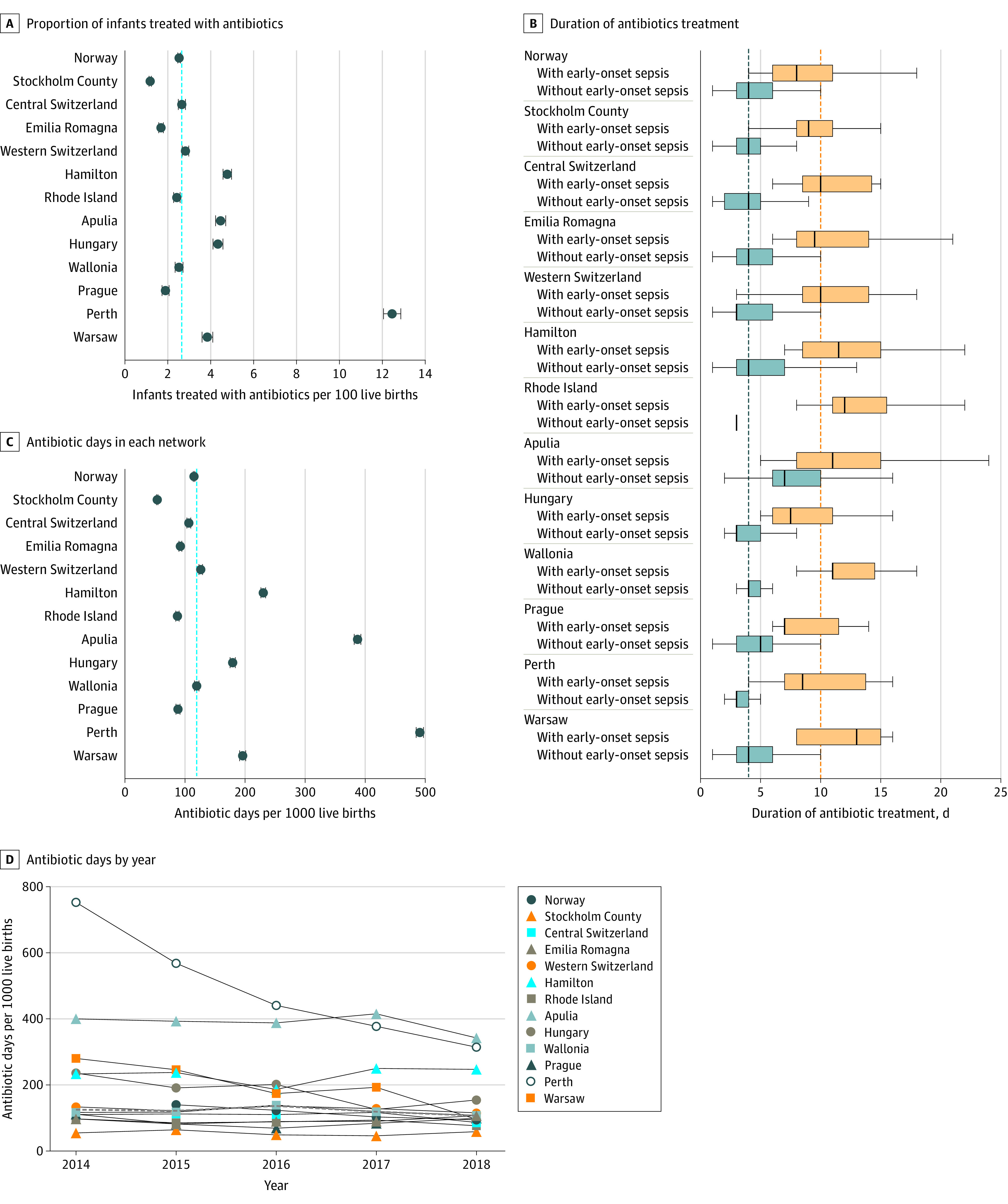
Burden of Treatment A, Proportion of infants treated with antibiotics (error bars denote 95% CIs) in each network. B, Duration of antibiotic treatment (error bars denote 95% CIs) in infants with early-onset sepsis and in infants without early-onset sepsis. The 239 patients who died were not included in this graph. C, Number of antibiotic days per 1000 live births (error bars denote 95% CIs) in each network. D, Number of antibiotic days per 1000 live births by year for each network. The dashed lines represent the median of the 13 networks.

**Figure 2.**
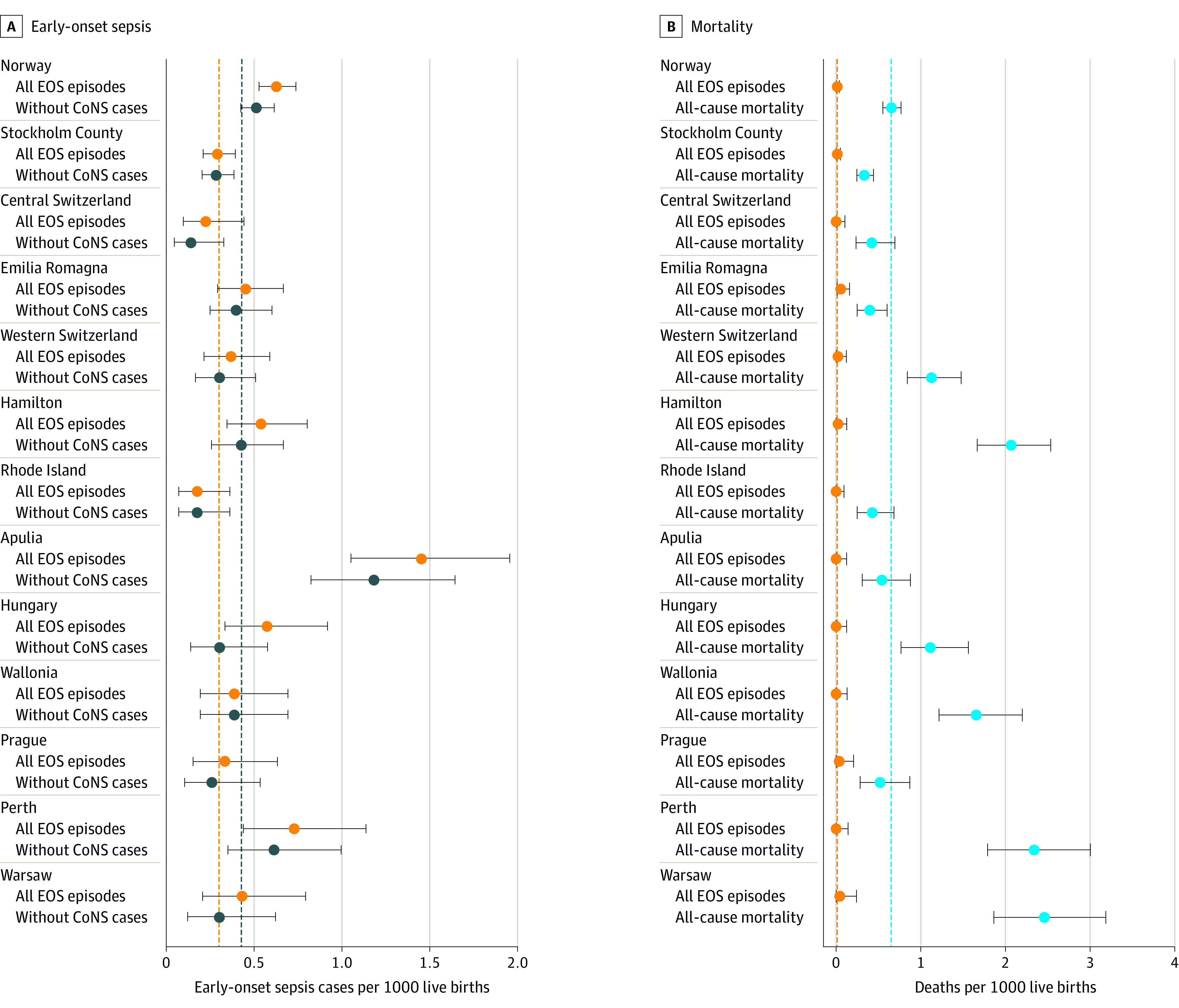
Burden of Disease A, Incidence of all culture-proven early-onset sepsis (EOS) episodes and incidence calculated without inclusion of coagulase-negative staphylococci (CoNS) cases in each network are shown (error bars denote 95% CIs). B, All-cause mortality and mortality in EOS cases per 1000 live births (error bars denote 95% CIs) in each network are shown. The dashed lines represent the median of the 13 networks.

**Figure 3.**
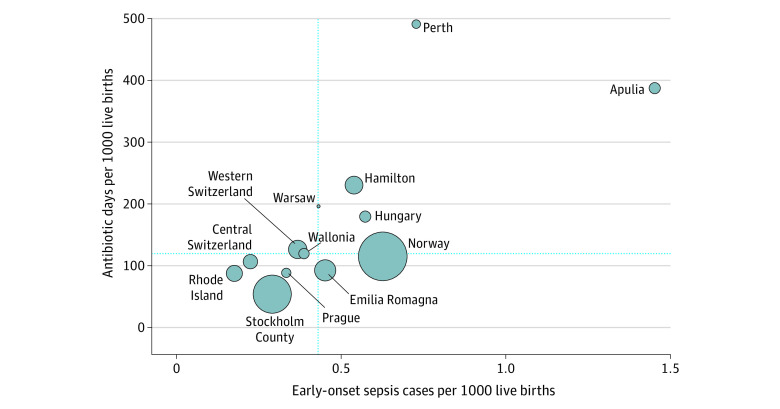
Burden of Treatment vs Burden of Disease Relationship between the incidence of early-onset sepsis and exposure to antibiotics in each network. The size of the bubbles represents the number of births. The dashed lines represent the median of the 13 networks.

Exposure to antibiotics, incidence of EOS, and all-cause mortality varied substantially across networks (Figure 1, Figure 2, and Table 1). The proportion of neonates treated with antibiotics ranged from 1.18% to 12.45% (a 9-fold difference). Three of the 13 networks reported treatment rates below 2%. Median duration of antibiotic treatment ranged from 8 to 12 days in infants with EOS and from 3 to 7 days in infants without proven EOS. The number of antibiotic days per 1000 live births ranged from 54 to 491. The incidence of EOS ranged from 0.18 to 1.45 cases per 1000 live births (an 8-fold difference), and the incidence of EOS without inclusion of CoNS cases ranged from 0.14 to 1.18 cases per 1000 live births. All-cause mortality was 0.33 to 2.46 per 1000 live births, and mortality in EOS cases ranged from 0% to 12%. Antibiotic exposure correlated with EOS incidence in different networks (Spearman *r* = 0.77; 95% CI, 0.39-0.93 for all EOS episodes; *r* = 0.69; 95% CI, 0.23-0.90 for EOS episodes without CoNS) (Figure 3 and eFigure 2 in Supplement 1).

Over time, when analyzing data without Norway (which provided only 2015-2018 data), the proportion of neonates treated with antibiotics decreased from 3.4% (95% CI, 3.3%-3.5%) in 2014 to 2.7% (95% CI, 2.6%-2.8%) in 2018, with no change in the duration of treatment (eFigure 3 in Supplement 1). This resulted in a decreased antibiotic exposure from 167 antibiotic days per 1000 live births (95% CI, 165-170 antibiotic days per 1000 live births) in 2014 to 127 antibiotic days per 1000 live births (95% CI, 125-129 antibiotic days per 1000 live births) in 2018. Incidence of EOS and all-cause and EOS-associated mortality did not change during the study period (eFigure 4 in Supplement 1).

In Stockholm County, Sweden, a large population-based network with the lowest exposure to antibiotics, the number of antibiotic days per 1000 live births was 50 (95% CI, 49-52 antibiotic days per 1000 live births) in neonates born in level I and II facilities and 63 antibiotic days per 1000 live births (95% CI, 61-65 antibiotic days per 1000 live births) in neonates born in level III and IV facilities (eTable 4 in Supplement 1). The incidence of EOS in Stockholm County was 0.25 case per 1000 live births (95% CI, 0.16-0.37 case per 1000 live births) in neonates born in level I and II facilities and 0.40 case per 1000 live births (95% CI, 0.23-0.64 case per 1000 live births) in neonates born in level III and IV facilities.

## Discussion

To our knowledge, this cross-sectional study is the largest international study on antibiotic exposure started in the first postnatal week in late-preterm and full-term neonates. Nearly 3% of neonates were started on antibiotics, resulting in an antibiotic exposure of 135 days per 1000 live births. The overall incidence of culture-proven EOS was 0.49 case per 1000 live births with a mortality rate of 3.20%. For each case of EOS, 58 neonates were started on antibiotics and 273 antibiotic days were administered. The burden of treatment varied up to 9-fold across the 13 networks, and the burden of disease varied up to 8-fold.

The burden of disease is associated with the incidence of EOS and sepsis-associated morbidity and mortality, which are inversely associated with gestational age.^19,20,21^ In Europe, the US, and Australia, the incidence of EOS in late-preterm and full-term neonates ranges between 0.13 and 0.95 per 1000 live births and has decreased over the last decades.^17,20,21,22,23,24^ As death from EOS has become a rare event in late-preterm and full-term neonates, studies analyzing several hundreds of thousands neonates are required to provide accurate estimates of EOS-associated mortality. Consistent with recent estimates, we observed an EOS-associated mortality rate of 3.20%.^17,21,25,26^

Precise data on antibiotic utilization are needed to improve antimicrobial prescription practices promoting quality of care and patient safety. Given the specificities of neonatal care and the importance of AMS in early life, relevant indicators are required to identify antibiotics utilization practices that should be promoted or avoided.^15^ Using the number of births rather than the number of hospitalizations as a denominator allows us to quantify antibiotic exposure at the population level. We described the burden of antibiotic treatment as the (1) proportion of all live births started on antibiotics, (2) duration of therapy, and (3) number of antibiotic days per 1000 live births. The proportion of neonates started on antibiotics in our study is at the low end of the up to 14% described in the literature.^11,17,24,27^ Three of the 13 networks reported treatment rates below 2% with the lowest rate of 1.18% in Stockholm County. To our knowledge, this is the lowest rate reported in literature. Most recent publications on the proportion of neonates treated with antibiotics reported on the impact of using the neonatal EOS calculator, a risk stratification and decision-making tool that recommends starting antibiotics when the estimated risk of EOS is 3 cases or more per 1000.^17^ Although the proportion of neonates started on antibiotics was reduced by this tool, the lowest rate of antibiotic exposure reported after implementing the EOS calculator is 3.0%.^11,17,27^ In our study, the only network using the EOS calculator decreased antibiotic treatment rates, but treatment rates after the intervention remained above all other networks. Seven networks used the serial physical examination approach, a strategy that has been associated with safe reduction of antibiotic use.^28,29,30^

Networks performed differently at minimizing the proportion of neonates started on antibiotics, limiting the duration of treatment in those without proven infection, and at preventing and/or diagnosing EOS. Networks with higher antibiotic exposure also had higher EOS incidence. This contrasts with findings from studies^24,31^ showing wide variations in antibiotic use across neonatal units but no correlation between the proportion of neonates exposed to antibiotics and the incidence of EOS at each site. Population-based networks include all neonates born in a given geographical area (comprising hospital and non–hospital-based delivery units and home births). In contrast, hospital-based networks include only neonates born at participating hospitals and are, therefore, not representative of a region or a country. Moreover, comparisons between hospital-based networks are limited by the specific patients’ case mix at each institution that may influence the risk of EOS and antibiotic prescription patterns. In our study, tertiary care hospitals (levels III and IV) which are characterized by higher patient acuity and volume, were overrepresented in hospital-based networks. To address these limitations, we performed analyses stratifying neonates born in Stockholm County (a large population-based network with the lowest exposure to antibiotics and the lowest all-cause mortality) by the level of care at the place of birth. The burden of disease and the burden of treatment were higher for neonates born in level III and IV facilities compared with level I and II facilities. This is not unexpected given the mission of level III and IV facilities to take care of the most vulnerable and most ill patients. However, in Stockholm County, the proportion of neonates treated with antibiotics and number of antibiotic days per 1000 live births was lower both in level I and II and in level III and IV facilities compared with all other networks. This indicates that antibiotic exposure can be minimized even in high-risk tertiary care facilities, without increasing all-cause mortality.

We observed substantial heterogeneity among networks regarding the strategies used to prevent EOS and manage newborns at risk of EOS^32^ but could not identify a common pattern of approaches associated with a lower burden of disease and/or treatment. Therefore, we are not able to generate a hypothesis regarding which strategy may work best. We speculate that education of staff, leadership, and AMS culture might have an important impact on clinical decision-making.^2,12,13,33,34^

Given the relatively low incidence of EOS and sepsis-associated mortality in late-preterm and full-term newborns, the burden of treatment is disproportionate to the burden of disease. This imbalance is bigger in full-term neonates, who have a lower sepsis-related mortality than late-preterm neonates.^21,23^ Culture-proven EOS accounted for 1.9% of all-cause mortality, and all-cause mortality was more frequent than EOS. This is in line with literature showing that perinatal asphyxia and congenital malformations cause a greater number of deaths in late-preterm and full-term neonates than sepsis.^35^

Large cohorts reporting on the burden of disease along with the burden of treatment are scarce. Previous studies^11,17,36^ focused mainly on 1 dimension and/or did not provide important baseline information, such as the proportion of all live-born neonates treated with antibiotics and/or the duration of treatment. To facilitate benchmarking and guide clinicians regarding AMS and future research, we propose a new minimal standard including 7 key indicators for reporting data on EOS and antibiotic use: (1) incidence of culture-proven EOS per 1000 live births, (2) EOS-associated mortality rate, (3) proportion of neonates started on antibiotics per 100 live births, (4) duration of antibiotic therapy, and (5) number of antibiotic days per 1000 live births, and the whole study population needs to be described by (6) gestational age and (7) all-cause mortality.

### Limitations

Although the strengths of our study are grounded in the large, international, high-quality data set promoting new standards to quantify the burden of disease along with the burden of treatment, our study has limitations. Owing to the retrospective design, we could not collect data on prenatal exposure to antibiotics, risk factors for EOS, biomarkers, and long-term morbidity. In addition, data on management strategies are descriptive, whereas clinical practice may substantially deviate from guidelines. The heterogeneity between and within networks and the selection bias of participating networks limits the generalizability of our findings. Population-based networks are representative of a region or a country, but hospital-based networks depend on the level of care and case selection at each site. The difficulty in distinguishing CoNS-related infections from contamination is a limitation that we addressed by showing results with and without CoNS-related infections. There is no clear-cut difference in the definition between perinatal (EOS) vs nosocomial (late-onset) infections. Including neonates started on antibiotics within the first postnatal week leads to some overlap between the 2 conditions. From an AMS perspective, this has the advantage of capturing all antibiotic courses started within a well-defined period.^2,4^ Given that more than 95% of antibiotic courses initiated within the first postnatal week were started within the first 3 days, it is unlikely that the choice of a 7-day rather than a 3-day cutoff affected our results.

## Conclusions

The burden of treatment is considerable in comparison to the burden of disease, and there are wide variations internationally. We defined a set of indicators reporting on both dimensions to facilitate benchmarking and guide AMS programs and future research. A neonatal antibiotic treatment rate less than or equal to 1% appears achievable. Decreasing unwarranted neonatal antibiotic exposure may help reduce the risk of antimicrobial resistance and preserve the developing microbiome to improve long-term health.

## Notes

Supplement 1. **eAppendix.** Sample Size Calculation **eTable 1.** Description of Networks **eTable 2.** Strategies to Prevent and Treat EOS and Main Outcomes in the 13 Networks **eTable 3.** Pathogens Identified in Blood and Cerebrospinal Fluid Cultures of Infants With Early-Onset Sepsis **eTable 4.** Main Outcomes in Infants Born in Level I-II vs III-IV Facilities From Stockholm County **eFigure 1.** Postnatal Age at Antibiotics Start **eFigure 2.** Relationship Between the Incidence of Early-Onset Sepsis Without Coagulase Negative Staphylococci and Exposure to Antibiotics **eFigure 3.** Burden of Treatment Over Time **eFigure 4.** Burden of Disease Over Time
eReferences

Supplement 2.
AENEAS Study Group Members

## References

[1] Versporten A, Zarb P, Caniaux I, ; Global-PPS network. Antimicrobial consumption and resistance in adult hospital inpatients in 53 countries: results of an internet-based global point prevalence survey. Lancet Glob Health. 2018;6(6):e619-e629. doi:10.1016/S2214-109X(18)30186-4 29681513

[2] Prusakov P, Goff DA, Wozniak PS, ; Global NEO-ASP Study Group. A global point prevalence survey of antimicrobial use in neonatal intensive care units: the no-more-antibiotics and resistance (NO-MAS-R) study. EClinicalMedicine. 2021;32:100727. doi:10.1016/j.eclinm.2021.100727 33554094 PMC7848759

[3] Hufnagel M, Versporten A, Bielicki J, Drapier N, Sharland M, Goossens H; ARPEC Project Group. High rates of prescribing antimicrobials for prophylaxis in children and neonates: results from the Antibiotic Resistance and Prescribing in European Children Point Prevalence Survey. J Pediatric Infect Dis Soc. 2019;8(2):143-151. doi:10.1093/jpids/piy019 29579259

[4] World Health Organization. World Health Assembly 69. Global action plan on antimicrobial resistance: options for establishing a global development and stewardship framework to support the development, control, distribution and appropriate use of new antimicrobial medicines, diagnostic tools, vaccines and other interventions—report by the Secretariat. 2016. Accessed April 5, 2022. https://apps.who.int/iris/handle/10665/252682

[5] Fjalstad JW, Esaiassen E, Juvet LK, van den Anker JN, Klingenberg C. Antibiotic therapy in neonates and impact on gut microbiota and antibiotic resistance development: a systematic review. J Antimicrob Chemother. 2018;73(3):569-580. doi:10.1093/jac/dkx426 29182785

[6] Antimicrobial Resistance Collaborators. Global burden of bacterial antimicrobial resistance in 2019: a systematic analysis. Lancet. 2022;399(10325):629-655. doi:10.1016/S0140-6736(21)02724-0 35065702 PMC8841637

[7] Stiemsma LT, Michels KB. The role of the microbiome in the developmental origins of health and disease. Pediatrics. 2018;141(4):e20172437. doi:10.1542/peds.2017-2437 29519955 PMC5869344

[8] Sourour W, Sanchez V, Sourour M, . The association between prolonged antibiotic use in culture negative infants and length of hospital stay and total hospital costs. Am J Perinatol. Published online May 11, 2021. doi:10.1055/s-0041-1729560 33975363

[9] Stark A, Smith PB, Hornik CP, . Medication use in the neonatal intensive care unit and changes from 2010 to 2018. J Pediatr. 2022;240:66-71.e4. doi:10.1016/j.jpeds.2021.08.075 34481808 PMC9394450

[10] Benitz WE, Achten NB. Finding a role for the neonatal early-onset sepsis risk calculator. EClinicalMedicine. 2020;19:100255. doi:10.1016/j.eclinm.2019.100255 32140673 PMC7046501

[11] Achten NB, Klingenberg C, Benitz WE, . Association of use of the neonatal early-onset sepsis calculator with reduction in antibiotic therapy and safety: a systematic review and meta-analysis. JAMA Pediatr. 2019;173(11):1032-1040. doi:10.1001/jamapediatrics.2019.2825 31479103 PMC6724419

[12] Klingenberg C, Kornelisse RF, Buonocore G, Maier RF, Stocker M. Culture-negative early-onset neonatal sepsis: at the crossroad between efficient sepsis care and antimicrobial stewardship. Front Pediatr. 2018;6:285. doi:10.3389/fped.2018.00285 30356671 PMC6189301

[13] Cantey JB, Patel SJ. Antimicrobial stewardship in the NICU. Infect Dis Clin North Am. 2014;28(2):247-261. doi:10.1016/j.idc.2014.01.005 24857391

[14] Araujo da Silva AR, Marques A, Di Biase C, . Effectiveness of antimicrobial stewardship programmes in neonatology: a systematic review. Arch Dis Child. 2020;105(6):563-568. doi:10.1136/archdischild-2019-318026 32156697

[15] Flannery DD, Horbar JD. Metrics of neonatal antibiotic use. Semin Perinatol. 2020;44(8):151329. doi:10.1016/j.semperi.2020.151329 33158602

[16] Fitchett EJA, Seale AC, Vergnano S, ; SPRING (Strengthening Publications Reporting Infection in Newborns Globally) Group. Strengthening the Reporting of Observational Studies in Epidemiology for Newborn Infection (STROBE-NI): an extension of the STROBE statement for neonatal infection research. Lancet Infect Dis. 2016;16(10):e202-e213. doi:10.1016/S1473-3099(16)30082-2 27633910

[17] Kuzniewicz MW, Puopolo KM, Fischer A, . A quantitative, risk-based approach to the management of neonatal early-onset sepsis. JAMA Pediatr. 2017;171(4):365-371. doi:10.1001/jamapediatrics.2016.4678 28241253

[18] American Academy of Pediatrics Committee on Fetus And Newborn. Levels of neonatal care. Pediatrics. 2012;130(3):587-597. doi:10.1542/peds.2012-1999 22926177

[19] Giannoni E, Agyeman PKA, Stocker M, ; Swiss Pediatric Sepsis Study. Neonatal sepsis of early onset, and hospital-acquired and community-acquired late onset: a prospective population-based cohort study. J Pediatr. 2018;201:106-114.e4. doi:10.1016/j.jpeds.2018.05.048 30054165

[20] Schrag SJ, Farley MM, Petit S, . Epidemiology of invasive early-onset neonatal sepsis, 2005 to 2014. Pediatrics. 2016;138(6):e20162013. doi:10.1542/peds.2016-2013 27940705

[21] Stoll BJ, Puopolo KM, Hansen NI, ; Eunice Kennedy Shriver National Institute of Child Health and Human Development Neonatal Research Network. Early-onset neonatal sepsis 2015 to 2017, the rise of *Escherichia coli*, and the need for novel prevention strategies. JAMA Pediatr. 2020;174(7):e200593. doi:10.1001/jamapediatrics.2020.0593 32364598 PMC7199167

[22] Cailes B, Kortsalioudaki C, Buttery J, ; neonIN Network. Epidemiology of UK neonatal infections: the neonIN Infection Surveillance Network. Arch Dis Child Fetal Neonatal Ed. 2018;103(6):F547-F553. doi:10.1136/archdischild-2017-313203 29208666

[23] Braye K, Foureur M, de Waal K, Jones M, Putt E, Ferguson J. Epidemiology of neonatal early-onset sepsis in a geographically diverse Australian health district 2006-2016. PLoS One. 2019;14(4):e0214298. doi:10.1371/journal.pone.0214298 30958832 PMC6453454

[24] Schulman J, Benitz WE, Profit J, . Newborn antibiotic exposures and association with proven bloodstream infection. Pediatrics. 2019;144(5):e20191105. doi:10.1542/peds.2019-1105 31641017

[25] Escobar GJ, Puopolo KM, Wi S, . Stratification of risk of early-onset sepsis in newborns ≥ 34 weeks’ gestation. Pediatrics. 2014;133(1):30-36. doi:10.1542/peds.2013-1689 24366992 PMC4079292

[26] Mundal HS, Rønnestad A, Klingenberg C, Stensvold HJ, Størdal K. Antibiotic use in term and near-term newborns. Pediatrics. 2021;148(6):e2021051339. doi:10.1542/peds.2021-051339 34814187

[27] Goel N, Cannell S, Davies G, . Implementation of an adapted Sepsis Risk Calculator algorithm to reduce antibiotic usage in the management of early onset neonatal sepsis: a multicentre initiative in Wales, UK. Arch Dis Child Fetal Neonatal Ed. 2022;107(3):303-310. doi:10.1136/archdischild-2020-321489 34551917

[28] Berardi A, Bedetti L, Spada C, Lucaccioni L, Frymoyer A. Serial clinical observation for management of newborns at risk of early-onset sepsis. Curr Opin Pediatr. 2020;32(2):245-251. doi:10.1097/MOP.0000000000000864 31851052

[29] Vatne A, Klingenberg C, Øymar K, Rønnestad AE, Manzoni P, Rettedal S. Reduced antibiotic exposure by serial physical examinations in term neonates at risk of early-onset sepsis. Pediatr Infect Dis J. 2020;39(5):438-443. doi:10.1097/INF.0000000000002590 32301920

[30] Duvoisin G, Fischer C, Maucort-Boulch D, Giannoni E. Reduction in the use of diagnostic tests in infants with risk factors for early-onset neonatal sepsis does not delay antibiotic treatment. Swiss Med Wkly. 2014;144:w13981. doi:10.4414/smw.2014.13981 24964177

[31] Schulman J, Dimand RJ, Lee HC, Duenas GV, Bennett MV, Gould JB. Neonatal intensive care unit antibiotic use. Pediatrics. 2015;135(5):826-833. doi:10.1542/peds.2014-3409 25896845

[32] van Herk W, el Helou S, Janota J, . Variation in current management of term and late-preterm neonates at risk for early-onset sepsis: an international survey and review of guidelines. Pediatr Infect Dis J. 2016;35(5):494-500. doi:10.1097/INF.0000000000001063 26766143

[33] Steinmann KE, Lehnick D, Buettcher M, . Impact of empowering leadership on antimicrobial stewardship: a single center study in a neonatal and pediatric intensive care unit and a literature review. Front Pediatr. 2018;6:294. doi:10.3389/fped.2018.00294 30370263 PMC6194187

[34] Satterfield J, Miesner AR, Percival KM. The role of education in antimicrobial stewardship. J Hosp Infect. 2020;105(2):130-141. doi:10.1016/j.jhin.2020.03.028 32243953

[35] Grünebaum A, McCullough LB, Arabin B, Dudenhausen J, Orosz B, Chervenak FA. Underlying causes of neonatal deaths in term singleton pregnancies: home births versus hospital births in the United States. J Perinat Med. 2017;45(3):349-357. doi:10.1515/jpm-2016-0200 27754969

[36] Stocker M, van Herk W, El Helou S, ; NeoPInS Study Group. Procalcitonin-guided decision making for duration of antibiotic therapy in neonates with suspected early-onset sepsis: a multicentre, randomised controlled trial (NeoPIns). Lancet. 2017;390(10097):871-881. doi:10.1016/S0140-6736(17)31444-7 28711318

