# Supplementary material for: Analysis of Antibiotic Exposure and Early-Onset Neonatal Sepsis in Europe, North America, and Australia
Source: JAMA Netw Open. 2022 Nov 23;5(11):e2243691. doi: 10.1001/jamanetworkopen.2022.43691 (PMC9685486; doi:10.1001/jamanetworkopen.2022.43691)
Supplement: Supplement 1. — eAppendix. Sample Size Calculation eTable 1. Description of Networks eTable 2. Strategies to Prevent and Treat EOS and Main Outcomes in the 13 Networks eTable 3. Pathogens Identified in Blood and Cerebrospinal Fluid Cultures of Infants With Early-Onset Sepsis eTable 4. Main Outcomes in Infants Born in Level I-II vs III-IV Facilities From Stockholm County eFigure 1. Postnatal Age at Antibiotics Start eFigure 2. Relationship Between the Incidence of Early-Onset Sepsis Without Coagulase Negative Staphylococci and Exposure to Antibiotics eFigure 3. Burden of Treatment Over Time eFigure 4. Burden of Disease Over Time eReferences [file jamanetwopen-e2243691-s001.pdf]

Supplemental Online Content

Giannoni E, Dimopoulou V, Klingenberg C, et al; AENEAS Study Group. Analysis of antibiotic exposure and early-onset neonatal sepsis in Europe, North America, and Australia. *JAMA Netw Open*. 2022;5(11):e2243691. doi:10.1001/jamanetworkopen.2022.43691

eAppendix. Sample Size Calculation

eTable 1. Description of Networks

eTable 2. Strategies to Prevent and Treat EOS and Main Outcomes in the 13 Networks

eTable 3. Pathogens Identified in Blood and Cerebrospinal Fluid Cultures of Infants With Early-Onset Sepsis

eTable 4. Main Outcomes in Infants Born in Level I-II vs III-IV Facilities From Stockholm County

eFigure 1. Postnatal Age at Antibiotics Start

eFigure 2. Relationship Between the Incidence of Early-Onset Sepsis Without Coagulase Negative Staphylococci and Exposure to Antibiotics

eFigure 3. Burden of Treatment Over Time

eFigure 4. Burden of Disease Over Time

eReferences

This supplemental material has been provided by the authors to give readers additional information about their work.

© 2022 Giannoni E et al. *JAMA Network Open.*

eAppendix. Sample Size Calculation

Sample size calculation was made by the Clinical Trial Unit of the University Hospital of Lausanne, Switzerland. The approach was to include enough newborns per network to be able to calculate the proportion of infants treated with antibiotics in relation to the incidence of early-onset sepsis with an adequate level of precision. Based on a review of the literature, we estimated that the proportion of proven infection would be around 1.25% (1/80) among infants treated with antibiotics. Considering that ± 1% precision around a proportion is acceptable, we calculated that the minimum sample size per network would be 475 infants treated with antibiotics. To calculate the minimal number of births that should be included in each network, we considered that 2% of infants receive antibiotics within the first postnatal week (lower end of reported data). The minimal sample size was estimated at 475*(100/2) = 23750 live births.

eTable 1. Description of Networks

Network	Country	Type of network[a]	Region, hospitals
Norway	Norway	Population-based data	Whole country
Stockholm County	Sweden	Population-based data	County
Central Switzerland	Switzerland	Population-based data	Canton Luzern, Uri, Schwytz, Unterwald
Emilia Romagna	Italy	4 level ≥ III hospitals	Azienda Ospedaliero-Universitaria Policlinico, Modena<br>Azienda Ospedaliero-Universitaria Sant'Orsola, Bologna<br>Ospedale Maggiore, Bologna<br>Ospedale M Bufalini, Cesena
Western Switzerland	Switzerland	3 level ≥ III, 1 level II hospitals	University Hospital of Geneva<br>Lausanne University Hospital<br>Inselspital, University Hospital of Bern<br>Hospital of Morges
Hamilton	Canada	1 level ≥ III, 2 level II hospitals	McMaster Children's Hospital, Hamilton<br>St Joseph's Healthcare Hamilton<br>Niagara Health, St. Catharines
Rhode Island	USA	1 level ≥ III hospital	Women & Infants Hospital of Rhode Island
Hungary	Hungary	2 level ≥ III hospitals	University Hospital of Szeged<br>Semmelweis University Hospital
Apulia	Italy	3 level ≥ III hospitals	Policlinico Bari<br>Ospedali Riuniti Foggia<br>Miulli
Wallonia	Belgium	2 level ≥ III hospitals	Grand Hôpital de Charleroi<br>CHC Montlegia
Prague	Czech Republic	1 level ≥ III, 1 level II hospital	Motol University Hospital, Prague<br>Thomayer Hospital, Prague
Perth	Australia	1 level ≥ III hospital	King Edward Memorial Hospital for Women, Perth
Warsaw	Poland	2 level ≥ III hospitals	Princess Anna Mazowiecka Hospital, Warsaw<br>Institute of Mother and Child, Warsaw

[a]The level of care of hospitals was defined according to the classification of the American Academy of Pediatrics [reference #1].

© 2022 Giannoni E et al. *JAMA Network Open.*

eTable 2. Strategies to Prevent and Treat EOS and Main Outcomes in the 13 Networks

Country, region	GBS Screening[a]	GBS IAP[b]	Obstetrical guidelines to prevent EOS	Use of laboratory tests[c]	Antibiotics for chorioamnionitis[d]	Serial physical examination[e]	EOS calculator[f]	Neonatal guidelines to diagnose and treat EOS	Antimicrobial stewardship program[g]	Incidence of EOS[h]	Antibiotic exposure[i]
Norway	-	-	National guidelines[j]	+	-	-	-	-	-	0.63 (0.53-0.74)	115 (114-116)
Sweden, Stockholm County	-	-	National guidelines[k]	+	-	-	-	National guidelines[q]	-	0.29 (0.21-0.39)	54 (53-55)
Italy, Emilia Romagna	+	+	CDC guidelines[l]	-	-	+	-	Regional guidelines[r]	+	0.45 (0.29-0.67)	92 (90-95)
Western Switzerland	+	+	-	-	-	+	-	National guidelines[s]	-/+[w]	0.37 (0.21-0.59)	126 (123-129)
Canada, Hamilton	+	+	National guidelines[m]	+	-	+	-	National guidelines[m]	-	0.54 (0.25-0.80)	230 (227-234)
USA, Rhode Island	+	+	-	+	-	-	-	-	+	0.18 (0.07-0.36)	88 (85-90)
Central Switzerland	+	+	-	-	-	+	-	National guidelines[s]	-	0.22 (0.10-0.44)	107 (103-110)
Hungary	+	+	National guidelines	+	+	-	-	National guidelines	-	0.57 (0.33-0.92)	180 (175-184)
Italy, Apulia	+	+	CDC guidelines[l]	+	+	-	-	AAP guidelines[t]	-	1.45 (1.05-1.96)	387 (382-393)
Belgium, Wallonia	+	+	-	+	-	-/+[o]	-	National guidelines[u]	-	0.39 (0.19-0.69)	120 (116-123)
Czech Republic, Prague	+	+	Local guidelines	+	-	+	-	Local guidelines	+	0.33 (0.15-0.63)	88 (85-92)
Australia, Perth	+	+	Local guidelines	+	+	-	-/+[p]	Regional guidelines[v]	-/+[p]	0.73 (0.44-1.14)	491 (485-497)
Poland, Warsaw	+	+	National guidelines[n]	+	+	+	-	National guidelines	-	0.43 (0.21-0.79)	196 (191-201)

[a]Maternal screening for colonization with Group B Streptococcus (GBS)
[b]Intrapartum antibiotic prophylaxis (IAP) in case of maternal GBS colonization
[c]Use of laboratory tests (such as complete blood count and/or C reactive protein) to decide whether or not to initiate antibiotic treatment
[d]Starting antibiotics in all infants born from mothers with chorioamnionitis
[e]Use of written protocol for standardized serial physical examination and observation of asymptomatic infants from mothers with risk factors
[f]Use of the neonatal early-onset sepsis (EOS) calculator
[g]Defined as an organizational strategy implemented in neonatal units to promote appropriate use of antimicrobials through the implementation of evidence-based interventions
[h]Number of EOS cases (all cases) per 1000 livebirths (95% CI)
[i]Number of antibiotic days per 1000 livebirths (95% CI)
[j]National Norwegian guidelines [reference # 2]
[k]National Swedish guidelines [reference #3]
[l]Guidelines of the Centers for Disease Control and Prevention [reference #4]
[m]Guidelines of the Canadian Pediatric Society [reference #5]

© 2022 Giannoni E et al. *JAMA Network Open.*

[n]Guidelines of the Polish Society of Gynecologists and Obstetricians [reference #6]
[o]Serial physical examination was implemented in 2015 in one out of two hospitals.
[p]The EOS calculator was implemented in July 2016 and an antimicrobial stewardship program was implemented in 2017.
[q]National Swedish guidelines [reference #7]
[r]Regional guidelines [reference #8]
[s]Guidelines of the Swiss Society of Neonatology [reference #9]
[t]Guidelines of the American Academy of Pediatrics [reference #10]
[u]Belgian pediatric GBS guidelines [reference #11]
[v]Guidelines of the Government of Western Australia [reference #12]
[w]An antimicrobial stewardship program was implemented in one out of four hospitals.

© 2022 Giannoni E et al. *JAMA Network Open.*

eTable 3. Pathogens Identified in Blood and Cerebrospinal Fluid Cultures of Infants With Early-Onset Sepsis

Pathogens	Number of EOS episodes			Postnatal age at blood culture, days
	All EOS episodes (n = 375)	Fatal episodes (n = 12)	Non-fatal episodes (n = 363)	
Gram-positive bacteria	279 (74%)	6 (50%)	273 (75%)	
Group B streptococci	126 (34%)	2 (17%)	124 (34%)	0.5 (0-1)
Coagulase negative staphylococci	65 (17%)	1 (8%)	64 (18%)	1 (0-2)
Staphylococcus aureus	23 (6%)	0	23 (6%)	3 (1-4)
Viridans group streptococci	21 (6%)	0	21 (6%)	1 (0-2)
Enterococcus spp	10 (3%)	0	10 (3%)	1 (0-2)
Listeria monocytogenes	5 (1%)	1 (8%)	4 (1%)	0 (0-1)
Other Gram-positive bacteria	29 (8%)	2 (17%)	27 (7%)	1 (1-1)
Gram-negative bacteria	84 (22%)	6 (50%)	78 (21%)	
Escherichia coli	62 (17%)	5 (42%)	57 (16%)	1 (0-2)
Klebsiella spp	8 (2%)	1 (8%)	7 (2%)	3 (1-5)
Other Gram-negative bacteria	14 (4%)	0	14 (4%)	1 (0-1)
Candida albicans	2 (1%)	0	2 (1%)	2 (2-2)
Mixed pathogens	5 (1%)	0	5 (1%)	1 (0-2)
Unidentified pathogens	5 (1%)	0	5 (1%)	0 (0-1)

Categorical variables are presented as frequencies (%) and continuous variables as median (IQR). Column percentages are presented; percentages are based on available data for each variable.

© 2022 Giannoni E et al. *JAMA Network Open.*

eTable 4. Main Outcomes in Infants Born in Level I-II vs III-IV Facilities From Stockholm County

Outcomes	Level I-II facilities[a]	Level III-IV facilities[a]	Odds ratio or Median difference (95% CI)
Number of births	104091	40326	
Number of infants treated with antibiotics in all live births	1144 (1.1%)	557 (1.4%)	1.26 (1.14 - 1.40) [b]
Number of antibiotic days (days/1000 live births)	5250 (50)	2542 (63)	1.26 (1.14 - 1.39)[c]<br>1.25 (1.05 - 1.49)[d]
Number of EOS cases (per 1000 live births)	26 (0.25‰)	16 (0.40‰)	1.59 (0.80 - 3.08) [b]
Number of all-cause deaths (per 1000 live births)	24 (0.23‰)	24 (0.60‰)	2.58 (1.40 - 4.75)[b]
Duration of antibiotic treatment (days)	4 (3-6)	4 (3-5)	0 (0 - 0)[e]
Number of antibiotic days (per treated infant)	5250 (4.59)	2542 (4.56)	0.89 (0.75 - 1.07)[c]<br>0.99 (0.93 - 1.06)[d]
Number of EOS cases in infants treated with antibiotics	26 (2.3%)	16 (2.9%)	1.27 (0.63 - 2.49)[b]
Number of all-cause deaths in all infants treated with antibiotics	15 (1.2%)	17 (3.1%)	2.37 (1.10 - 5.13)[b]
Number of all-cause deaths in proven EOS cases	2 (7.7%)	0 (0.0%)	0 (0 - 3.15)[b]
Number of all-cause deaths in cases without proven infection	13 (1.2%)	17 (3.1%)	2.76 (1.25 - 6.22)[b]

[a]The level of care of hospitals was defined according to the classification of the American Academy of Pediatrics [reference #1].
[b]Odds ratio for Level III-IV facilities (compared to Level I-II facilities) with 95% confidence interval
[c]Odds ratio, derived from an ordered logistic regression, for Level III-IV facilities (compared to Level I-II facilities) with 95% confidence interval
[d]Incidence rate ratio, derived from a negative binomial regression, for Level III-IV facilities (compared to Level I-II facilities) with 95% confidence interval
[e]Median difference with 95% confidence interval

© 2022 Giannoni E et al. *JAMA Network Open.*

eFigure 1. Postnatal Age at Antibiotics Start

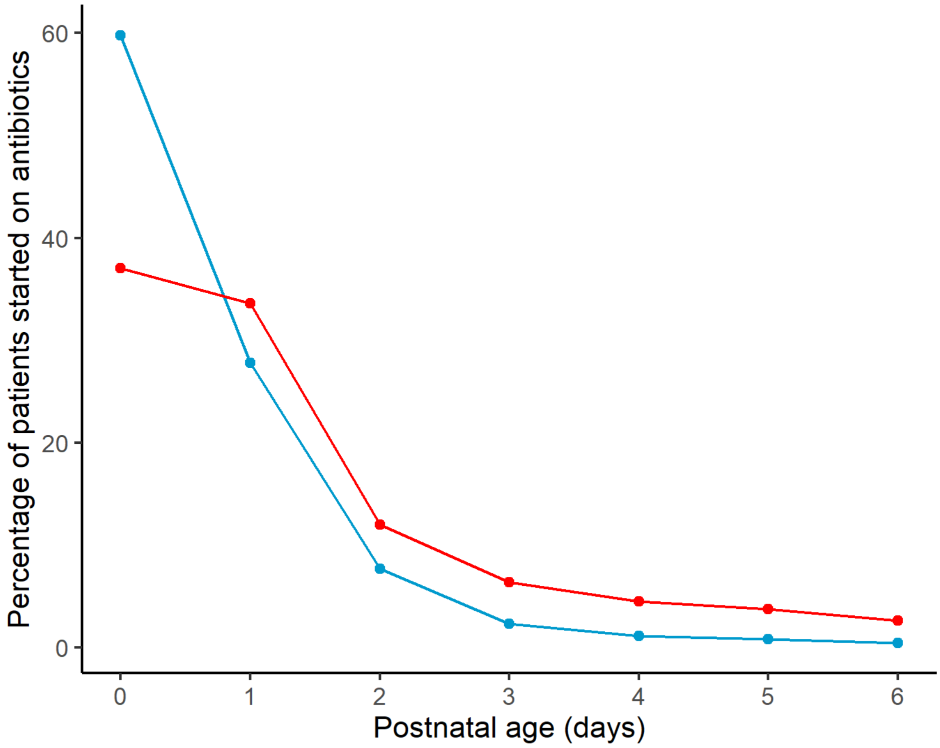

Number of infants started on antibiotics

EOS cases	139	126	45	24	17	14	10
Cases without proven EOS	12752	5927	1636	497	244	170	102

Postnatal age at antibiotic start in infants with (red dots, n = 375) and without (blue dots, n = 21328) early-onset sepsis (EOS).

© 2022 Giannoni E et al. *JAMA Network Open.*

eFigure 2. **Relationship Between the Incidence of Early-Onset Sepsis Without Coagulase Negative Staphylococci and Exposure to Antibiotics**

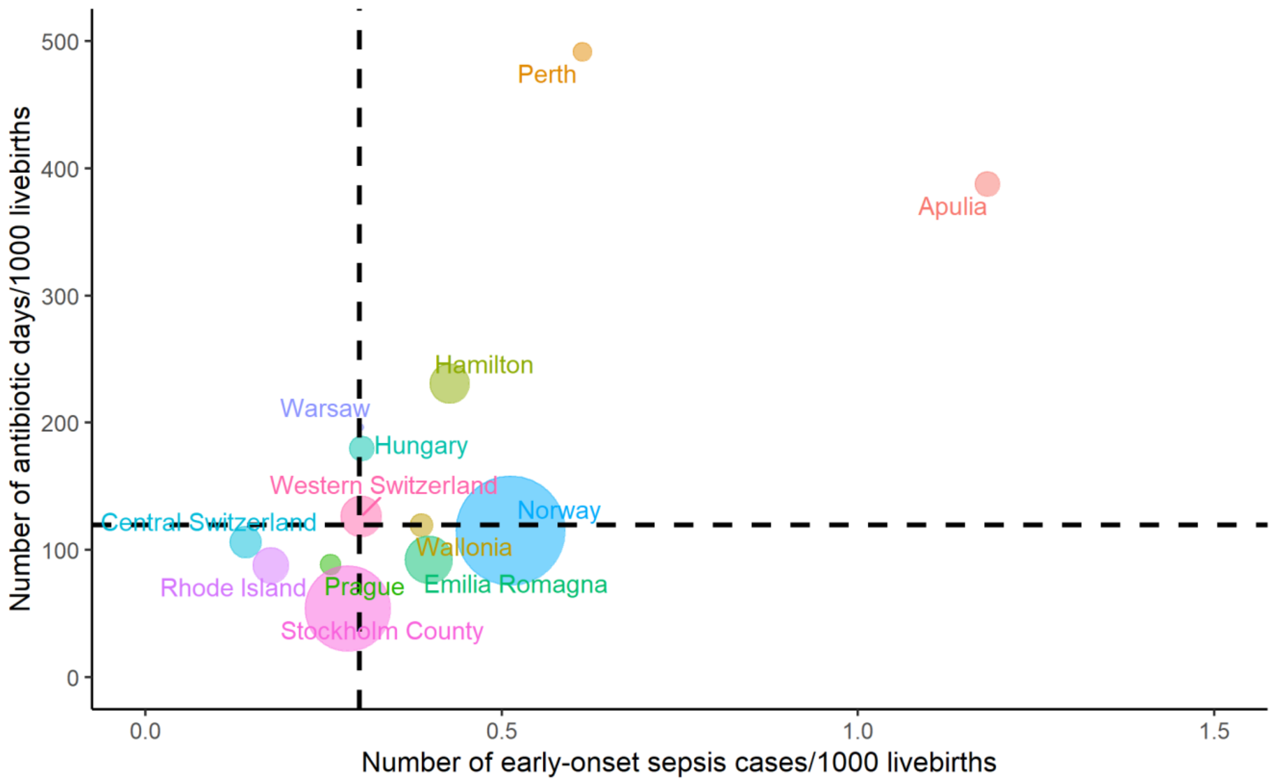

The size of the bubbles represents the number of births. The dotted lines represent the median of the 13 networks.

© 2022 Giannoni E et al. *JAMA Network Open.*

eFigure 3. Burden of Treatment Over Time

A

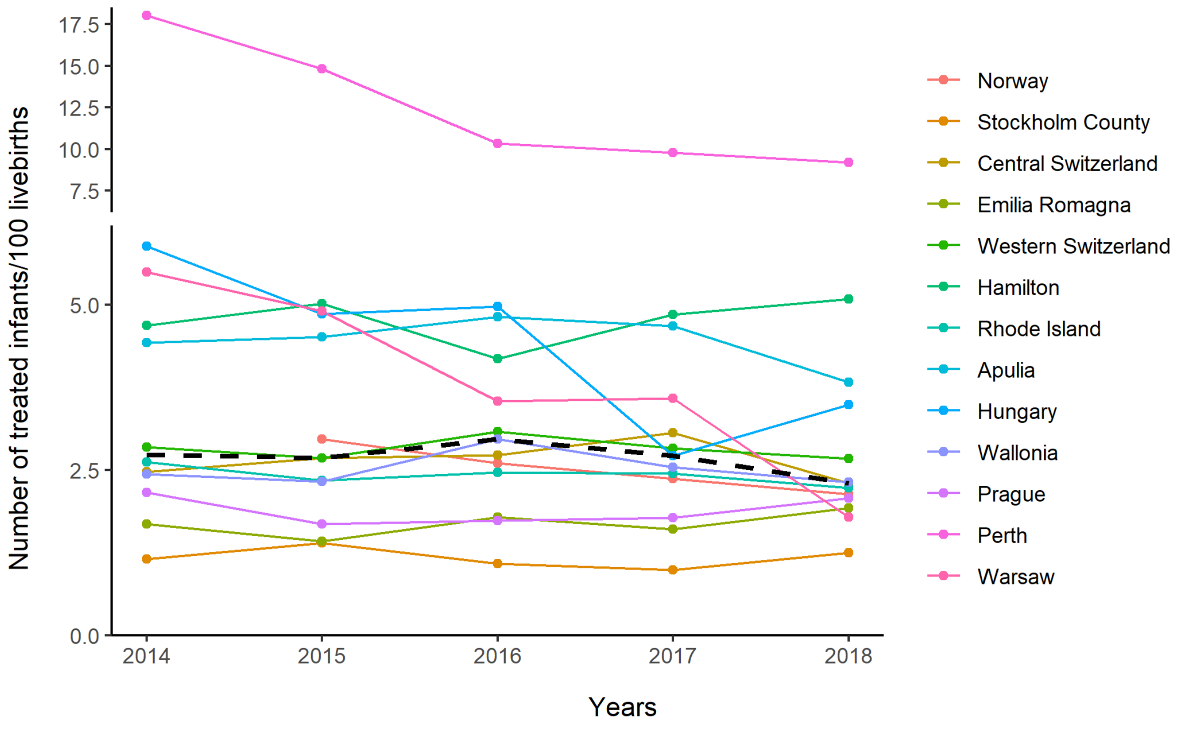

B

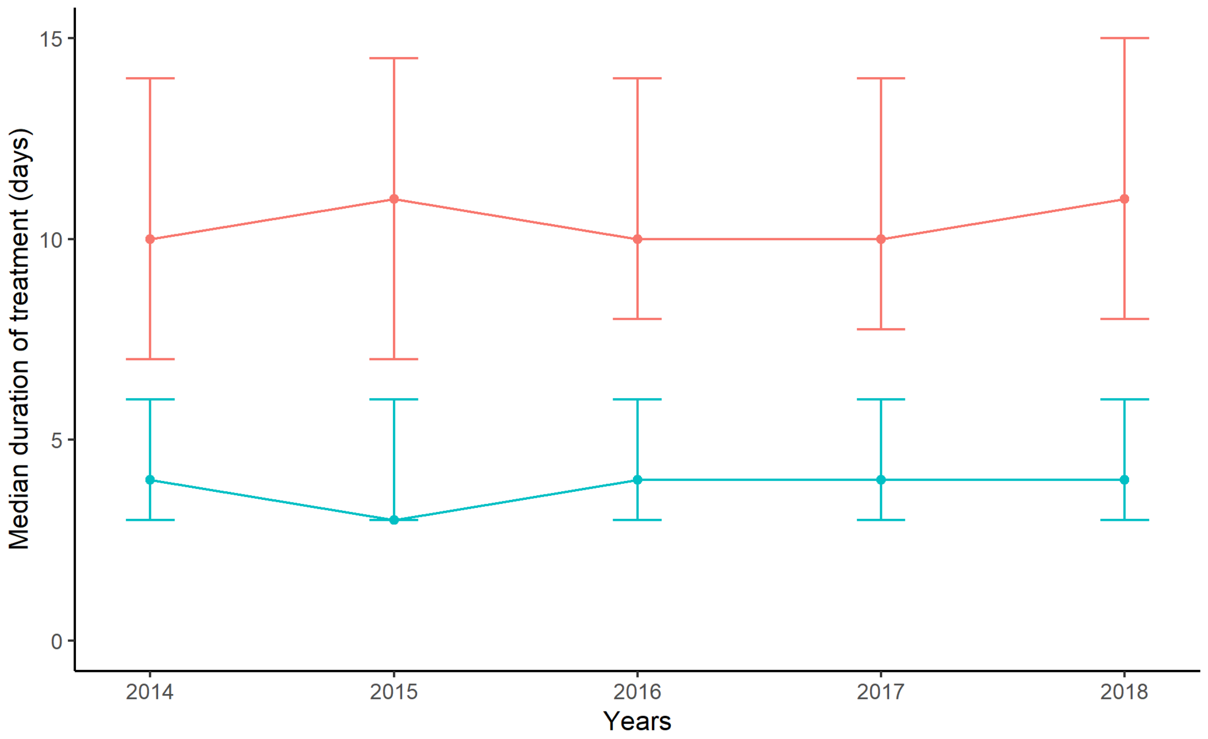

A) Proportion of infants treated with antibiotics by year in each network. The dashed line represent the median of the 13 networks. B) Duration of antibiotic treatment by year in infants with early-onset sepsis (in red), and in infants without early-onset sepsis (in blue). The median, 25th percentile, and 75th percentile are shown. Patients who died were not included this graph. Data on 529780/ 757979 births (69.9%) and 15936/21703 (73.4%) infants treated with antibiotics is presented, including 222/375 (59.2%) infants with proven EOS, and 15687/21328 (73.6%) infants without early-onset sepsis, as data from 2014 was not available for Norway.

© 2022 Giannoni E et al. *JAMA Network Open.*

eFigure 4. **Burden of Disease Over Time**

A

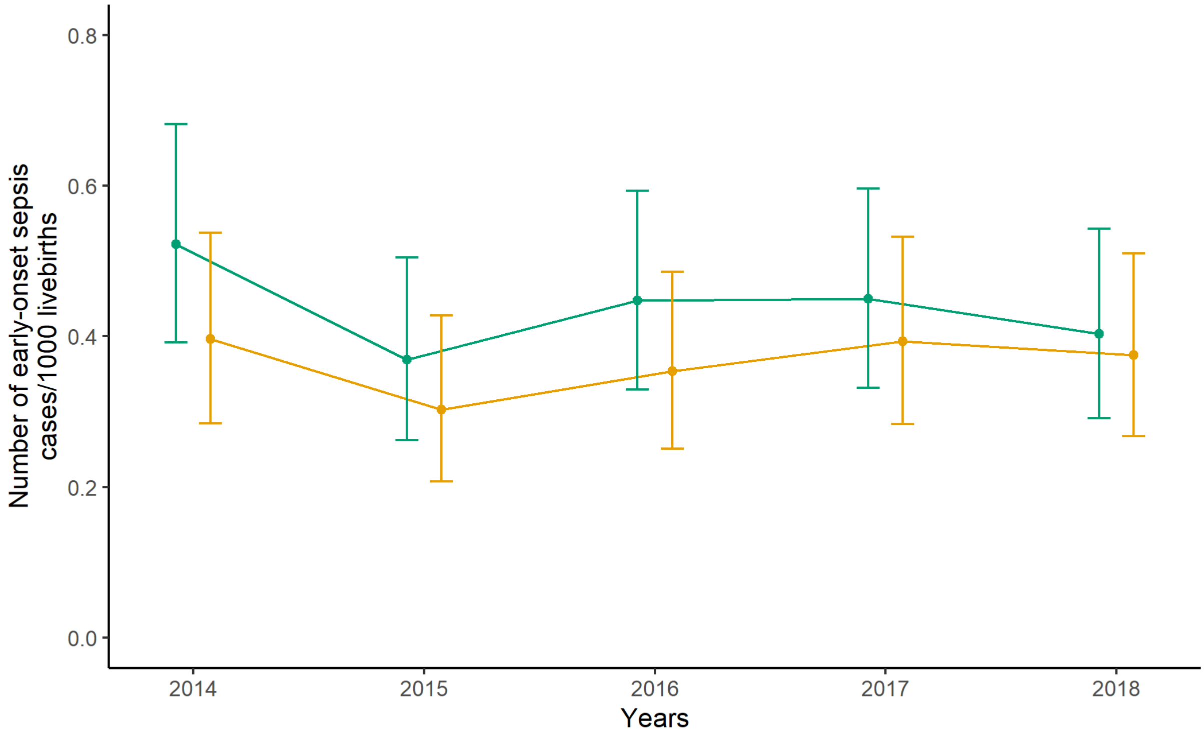

B

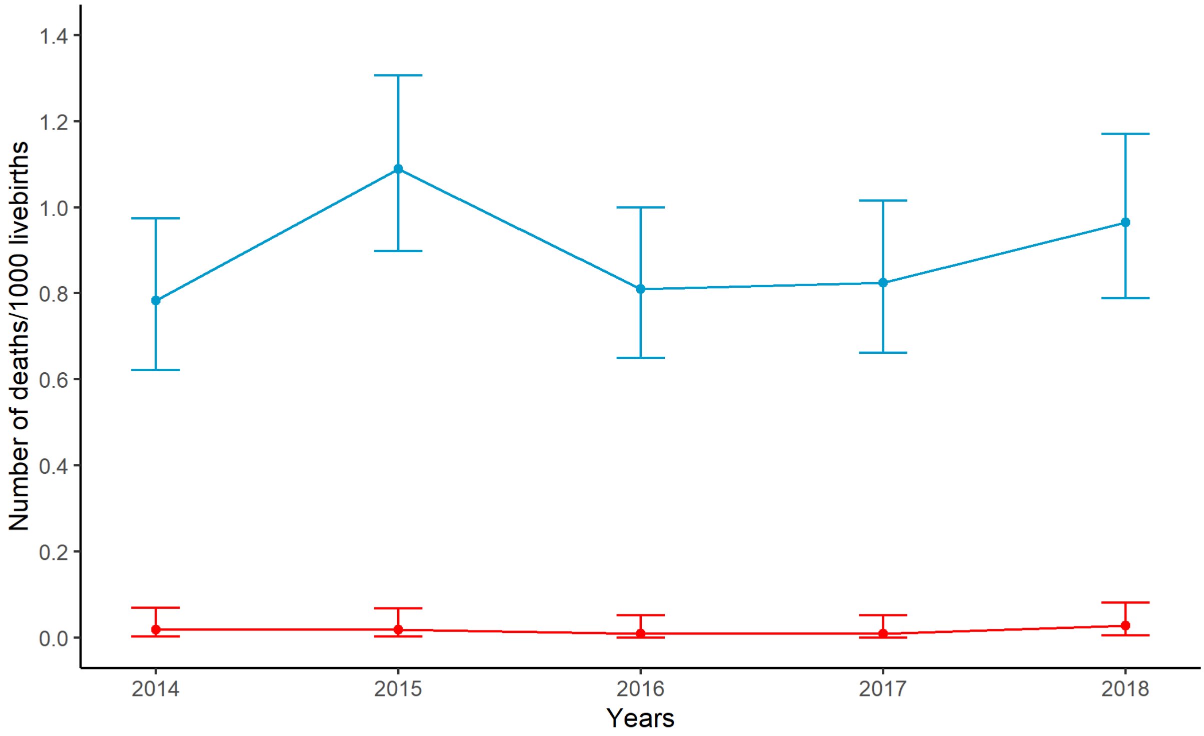

A) Incidence of early-onset sepsis by year and 95% CI ; incidence calculated with all culture-proven early-onset sepsis episodes is presented in green, incidence calculated without inclusion of coagulase-negative staphylococci cases is presented in orange. B) Mortality per 1000 livebirths by year and 95% CI ; all-cause mortality is presented in blue, and mortality in early-onset cases is presented in red. Data on 529780/ 757979 births (69.9%) and 15936/21703 (73.4%) infants treated with antibiotics is presented, including 222/375 (59.2%) infants with proven early-onset sepsis, and 15687/21328 (73.6%) infants without proven early-onset sepsis, as data from 2014 was not available for Norway.

© 2022 Giannoni E et al. *JAMA Network Open.*

eReferences

1. COMMITTEE ON FETUS AND NEWBORN, Barfield WD, Papile L-A, *et al.* Levels of Neonatal Care. *Pediatrics* 2012; 130: 587–97. doi: 10.1542/peds.2012-1999
2. Veileder i fødselshjelp (2020). https://www.legeforeningen.no/foreningsledd/fagmed/norsk-gynekologisk-forening/veiledere/veileder-i-fodselshjelp/ (accessed April 22, 2022)
3. Prevention av tidiga infektioner med grupp B-streptokocker (GBS) hos nyfödda. https://www.socialstyrelsen.se/globalassets/sharepoint-dokument/artikelkatalog/ovrigt/2008-130-7_20081307.pdf (accessed April 22, 2022)
4. Prevention of Perinatal Group B Streptococcal Disease. https://www.cdc.gov/mmwr/preview/mmwrhtml/rr5910a1.htm (accessed April 22, 2022)
5. Society CP. Management of term infants at increased risk for early onset bacterial sepsis | Canadian Paediatric Society. https://cps.ca/en/documents//position//management-infant-sepsis/ (accessed April 22, 2022)
6. Strona nie znaleziona | ptgin.pl. https://www.ptgin.pl/sites/default/files/page-2019/Wykrywanie%20nosicielstwa%20GBS%20u%20kobiet%20w%20ci%C4%85%C5%BCy%20i%20zapobiegania%20zaka%C5%BCeniom%20u%20noworodk%C3%B3w%20%28luty%202008%29.pdf (accessed April 22, 2022)
7. Neonatal sepsis – ny behandlingsrekommendation. Information från Läkemedelsverket 2013;24(3):15–25. https://www.lakemedelsverket.se/48e65c/globalassets/dokument/behandling-och-forskrivning/behandlingsrekommendationer/behandlingsrekommendation/lakemedel-vid-neonatal-sepsis-behandlingsrekommendation.pdf (accessed May 19, 2022)
8. Berardi A, Bedetti L, Spada C, Lucaccioni L, Frymoyer A. Serial clinical observation for management of newborns at risk of early-onset sepsis: *Current Opinion in Pediatrics* 2020; 32: 245–51. doi: 10.1097/MOP.0000000000000864
9. Stocker M, Berger C, McDougall J, Giannoni E. Recommendations for term and late preterm infants at risk for perinatal bacterial infection. *Swiss Med Wkly* 2013; published online Sept 19. doi:10.4414/smw.2013.13873
10. Polin RA, the COMMITTEE ON FETUS AND NEWBORN, Papile L-A, *et al.* Management of Neonates With Suspected or Proven Early-Onset Bacterial Sepsis. *Pediatrics* 2012; 129: 1006–15. doi: 10.1542/peds.2012-0541
11. Mahieu L, Langhendries J-P, Cossey V, De Praeter C, Lepage P, Melin P. Management of the neonate at risk for early-onset Group B streptococcal disease (GBS EOD): new paediatric guidelines in Belgium. *Acta Clinica Belgica* 2014; 69: 313–9. doi: 10.1179/2295333714Y.0000000054
12. CAHS | Child and Adolescent Health Service - Neonatology Postnatal Ward guidelines. https://cahs.health.wa.gov.au/For-health-professionals/Resources/Neonatology-Postnatal-Ward-guidelines (accessed April 22, 2022)

© 2022 Giannoni E et al. *JAMA Network Open.*
