# Supplementary material for: Analysis of Antibiotic Exposure and Early-Onset Neonatal Sepsis in Europe, North America, and Australia
Source: JAMA Netw Open. 2022 Nov 23;5(11):e2243691. doi: 10.1001/jamanetworkopen.2022.43691 (PMC9685486; doi:10.1001/jamanetworkopen.2022.43691)
Supplement: Supplement 2. — AENEAS Study Group Members [file jamanetwopen-e2243691-s002.pdf]

Supplemental Online Content: Nonauthor Collaborators
*First name, last name, and suffix (if applicable) are required and will appear in PubMed.

| *Group Name(s): AENEAS Study Group | | | | | | | |
|---|---|---|---|---|---|---|---|
| ***First Name and Middle Initial(s)** | ***Last Name** | ***Suffix (eg, Jr, III)** | Academic Degrees | Institution | Location (city, state/province, country) | Role or Contribution, eg, chair, principal investigator | Group (if more than 1 Group listed in the byline) and/or Subgroup (eg, Steering Committee) |
| Maria Grazia | Capretti | | | Neonatal Intensive Care Unit, IRCCS Azienda Ospedaliero-Universitaria di Bologna | Bologna, Italy | Participated in data collection | |
| Martina | Ceccoli | | | Neonatal Intensive Care Unit, Azienda Ospedaliero-Universitaria di Modena | Modena, Italy | Participated in data collection | |
| Morena | De Angelis | | | Neonatal Intensive Care Unit, Maggiore Hospital | Bologna, Italy | Participated in data collection | |
| Pietro | Drimaco | | | Neonatology and Neonatal Intensive Care Unit, Ecclesiastical General Hospital F. Miulli | Acquaviva delle Fonti, Italy | Participated in data collection | |
| Khalyane | Eap | | | University of Lausanne | Lausanne, Switzerland | Participated in data collection | |
| Zoe | el Helou | | | Division of Neonatology, Department of Pediatrics, McMaster Children's Hospital, McMaster University, Hamilton Health Sciences | Hamilton, Canada | Participated in data collection | |
| Rana | Esmaeilizand | | | Division of Neonatology, Department of Pediatrics, McMaster Children's Hospital, McMaster University, Hamilton Health Sciences | Hamilton, Canada | Participated in data collection | |
| Alessandra | Foglianese | | | Neonatologia e Terapia Intensiva Neonatale, University of Bari | Bari, Italy | Participated in data collection | |
| Carmelo | Geraci | | | Policlinico Riuniti di Foggia | Foggia, Italy | Participated in data collection | |

Supplemental Online Content: Nonauthor Collaborators
*First name, last name, and suffix (if applicable) are required and will appear in PubMed.

| ***First Name and Middle Initial(s)** | ***Last Name** | ***Suffix (eg, Jr, III)** | Academic Degrees | Institution | Location (city, state/province, country) | Role or Contribution, eg, chair, principal investigator | Group (if more than 1 Group listed in the byline) and/or Subgroup (eg, Steering Committee) |
|---|---|---|---|---|---|---|---|
| Bartłomiej | Grochowski | | | Department of Neonatology and Neonatal Intensive Care Medical University of Warsaw, Poland | Warsaw, Poland | Participated in data collection | |
| Stellan | Håkansson | | | The Swedish Neonatal Quality Register, Stockholm, Sweden and Department of Clinical Sciences, Pediatrics, Umeå University | Umeå, Sweden | Participated in data collection | |
| Sharandeep | Kaur | | | Department of Pediatrics, St Catharines General Hospital, Niagara Health | St Catharines, Canada | Participated in data collection | |
| Anne-Louise | Kollegger | | | MACCS Université Catholique de Louvain | Louvain, Belgium | Participated in data collection | |
| Frida | Oldendorff | | | Department of Neonatology, Karolinska University Hospital and Department of Clinical Science, Intervention and Technology (CLINTEC), Karolinska Institutet | Stockholm, Sweden | Participated in data collection | |
| Vittoria | Rizzo | | | Terapia Intensiva Neonatale Ospedale M.Bufalini Cesena | Cesena, Italy | Participated in data collection | |
| Arild E. | Rønnestad | | | Medical faculty, Institute for clinical medicine, University of Oslo | Oslo, Norway | Participated in data collection | |
| Damber | Shrestha | | | Neonatal Directorate, Child and Adolescent Health Service, King Edward Memorial Hospital | Perth, Western Australia | Participated in data collection | |
| Hans Jørgen | Stensvold | | | Department of Neonatal Intensive Care, Clinic of Paediatric and Adolescent Medicine, Oslo University Hospital | Oslo, Norway | Participated in data collection | |

Supplemental Online Content: Nonauthor Collaborators
*First name, last name, and suffix (if applicable) are required and will appear in PubMed.

| ***First Name and Middle Initial(s)** | ***Last Name** | ***Suffix (eg, Jr, III)** | Academic Degrees | Institution | Location (city, state/province, country) | Role or Contribution, eg, chair, principal investigator | Group (if more than 1 Group listed in the byline) and/or Subgroup (eg, Steering Committee) |
|---|---|---|---|---|---|---|---|
| Anaïs | Torregrossa | | | Department Mother-Woman-Child, Lausanne University Hospital and University of Lausanne | Lausanne, Switzerland | Participated in data collection | |
| Martin | Trefny | | | Department of Obstetrics and Gynecology, Neonatal unit, Motol University Hospital and Second Faculty of Medicine, Charles University | Prague, Czech Republic | Participated in data collection | |
| Kristyna | Zilinska | | | Department of Neonatology, Thomayer Hospital Prague | Prague, Czech Republic | Participated in data collection | |
| Aleksandra | Zwijacz | | | Department of Neonatology and Neonatl Intensive Care Medical University of Warsaw | Warsaw, Poland | Participated in data collection | |
